# Alternative Calibration of Cup Anemometers: A Way to Reduce the Uncertainty of Wind Power Density Estimation

**DOI:** 10.3390/s19092029

**Published:** 2019-04-30

**Authors:** Francisca Guerrero-Villar, Rubén Dorado-Vicente, Gustavo Medina-Sánchez, Eloísa Torres-Jiménez

**Affiliations:** Department of Mechanical and Mining Engineering; Universidad de Jaén, 23071 Jaén, Spain; mgvillar@ujaen.es (F.G.-V.); rdorado@ujaen.es (R.D.-V.); gmedina@ujaen.es (G.M.-S.)

**Keywords:** wind speed sensor, cup anemometer, quadratic regression uncertainty, wind dynamic pressure, wind power density, wind tunnel, anemometer calibration, pitot tube

## Abstract

This study presents a procedure to reduce the uncertainty of wind power density estimations, which is useful to improve the energy production predictions of wind farms. Power density is usually determined from the wind speed measured by a cup anemometer and the air density value (conventional procedure). An alternative procedure based on wind speed and dynamic pressure estimations provided by a cup anemometer is proposed. The dynamic pressure is obtained by means of a calibration curve that relates the anemometer rotation frequency and the dynamic pressure measured by a Pitot tube. The quadratic regression, used to define the calibration curve, and its uncertainty are both detailed. A comparison between the alternative procedure and the conventional one points out the advantage of the proposed alternative since results show a high reduction of the indirect measurement uncertainty of wind power density.

## 1. Introduction

Cup anemometer is a robust and reliable device. From the first cup anemometer, invented by Robinson [1], this device has been investigated and improved [2,3]. Current research provides analytical models and numerical studies related to its performance [4].

The use of cup anemometers is widely extended in fields such as meteorology and wind energy production. In the wind energy field, their use is increasing as the installed wind power capacity does [5]. In fact, wind speed metrology is key to assess the energy potential of wind turbines [6,7]. Accurate estimations of power curves are required to determine performance and the Annual Energy Production (AEP) of wind turbines [8]. 

The calibration of these devices is growing in importance, since regulations, regarding the determination of potential wind power production, establish a calibration of anemometers before and after each field data collection. In the wind energy field, power delivered by wind turbines is calculated from wind speed measurement. Even more, according to the international standard IEC 61400-12-1 [9], wind speed is needed to calculate the Annual Energy Production (AEP) and the power coefficient curve (*C_P_*):

(1) $$C_{P}=\frac{P}{\frac{1}{2}\cdot \;\rho \cdot S\cdot \;V^{3}}=\frac{P}{P_{w}\cdot \;S}\;,$$

where *P* is the electrical power produced by the wind turbine, *ρ* is the air density, *S* is the blade swept area, and *V* is the wind speed. *P_w_* is known as “wind power density” [10]: this magnitude allows the assessment of *C_P_*, which depends on the air conditions at the location of the turbine.

**Figure 1:**
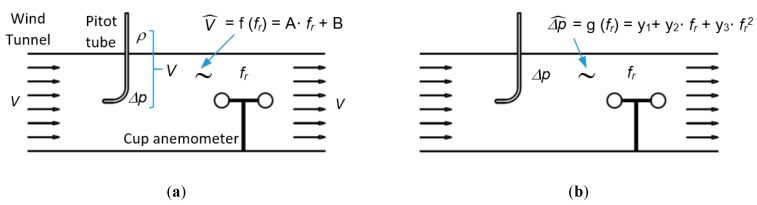
Calibration of a cup anemometer by means of a Pitot tube: (**a**) Conventional calibration: Air Speed Cup Anemometer (“AS-Cup Anemometer”), (**b**) Alternative calibration: Dynamic Pressure Cup Anemometer (“DYP-Cup Anemometer”).

In the calibration process of a cup anemometer, its rotational frequency *f_r_*, which provides the rotational speed, is usually related to the wind speed. Previous studies demonstrated that this is a linear relationship [11] (see Figure 1a), which is explained in detail by Pindado et al. [12]. Due to its reliability, the Measuring Network of Wind Energy Institutes (MEASNET) [13], the European Association of National Metrology Institutes EUROMET in their project titled “Comparison for Air speed Measurements” [14], and other authors such as Ghaemi-Nasab et al. [15] use the Pitot tube as the standard device in the cup anemometer calibration process.

Pitot tube provides the fluid flow velocity based on Bernoulli’s equation [16]. This device measures the difference between total and static pressure, which is call dynamic pressure and represents the fluid kinetic energy per unit volume. This difference is proportional to the square of fluid flow velocity multiplied by air density:

(2) $$\Delta p=p_{total}-p_{static}=\frac{1}{2}\cdot \;\rho \cdot \;V^{2}.$$

Since Pitot tube measures dynamic pressure instead of fluid velocity, it is proposed to calibrate the cup anemometer rotation frequency respect to the dynamic pressure. This calibration allows to estimate *P_w_* using the dynamic pressure and wind speed measured by a cup anemometer:

(3) $$P_{w}=\Delta p\;\cdot V.$$

Hereafter, the cup anemometer used to measure Δ*p* will be called as “Dynamic Pressure (DYP)-Cup Anemometer”, meanwhile if the anemometer is used to measure *V* it will be called as “Air Speed (AS)-Cup Anemometer” (see Figure 1).

The aerodynamic movement of the cup anemometer is mainly due to drag force [17], and this force is proportional to air dynamic pressure [18], which leads us to think that the relationship between air dynamic pressure and cup anemometer rotation frequency is quadratic (see Figure 1b). 

The targets of the present study are as follows: (a) show that *P_w_* can be obtained using a cup anemometer with a double calibration, so that *f_r_* provides Δ*p* and *V*, (b) determine the goodness and the uncertainty of the quadratic regression between the wind dynamic pressure and the cup anemometer rotation frequency, (c) quantify and compare the uncertainty of Δ*p* measurements, with the uncertainty of *V* measurements obtained according to the conventional method developed in annex F of IEC 61400-12-1 international standard [9], and (d) determine if the uncertainty of wind power density calculation decreases when the proposed method, based on dynamic pressure, is applied.

Because cup-anemometers are usually calibrated in wind tunnels, uncertainties calculated from the application of the conventional and proposed calibration are obtained using a wind tunnel. Note that in-field calibration has no clear advantages over conventional calibration [19]. Although it is possible to assess the measurement variability at a specific site, such as Aquila et al. [20] propose, it is difficult to compare those uncertainties to the conventional calibration method performed within a wind tunnel. In other studies, such as those with the target of determining the average flow velocity overestimation [21], cup anemometer uncertainty is analyzed under time-varying flow, nevertheless, the present study is focused on steady state flows because this is the appropriate procedure for the calibration of cup anemometers.

## 2. Materials and Methods

### 2.1. Experimental Set-up and Measurement Strategy

**Figure 2:**
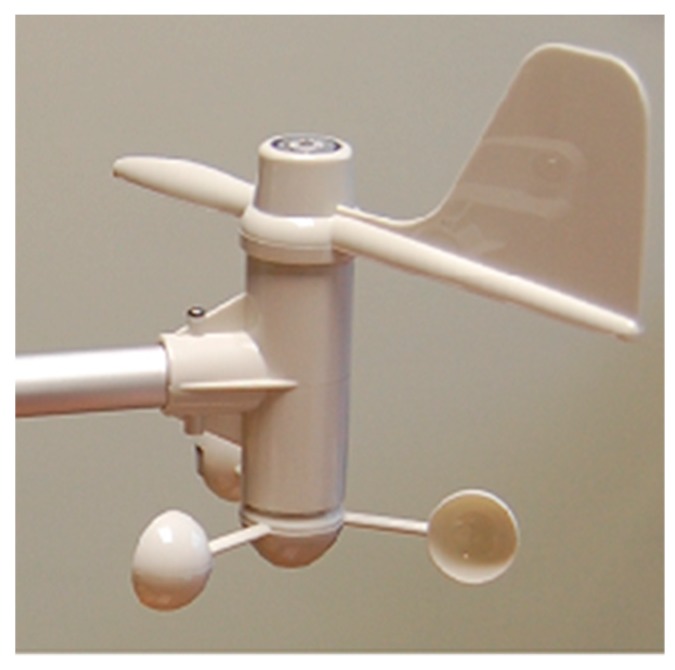
Cup anemometer used in the present study.

The experimental results presented in next sections are obtained by means of a cup anemometer taken from a commercial meteorological station Auriol model IAN 114435. This anemometer has three semi-spherical shape cups of 26 mm diameter each one, and 52 mm distance from the rotor axis to the center of the cups. This small cup anemometer with a rotor diameter of 130 mm (see Figure 2), has an appropriate size to be installed inside the tunnel available in the department of Mechanical and Mining Engineering of the University of Jaén. This cup anemometer has a vane, which has not been removed, because it does not disturb the experiment and it will be useful in future field applications.

**Figure 3:**
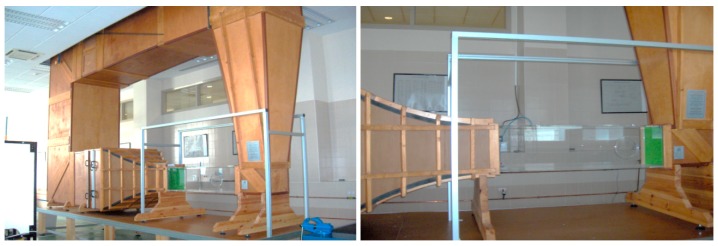
Wind tunnel used in the present study.

The cup anemometer was tested in a closed jet-type wind tunnel (see Figure 3), whose testing transversal area is 40 cm by 40 cm. The uniform main flow in the tunnel is produced by a 5 kW rotating fan with a variable speed drive engine, which provides a flow velocity between 1 m/s and 30 m/s. The wind tunnel turbulence levels are less than 2%, and the ratio of flow uniformity is high (within 0.2% for *x*, *y,* and *z* axis flows) thanks to several wire meshes, one honeycomb type filter and a convergent nozzle. Note that, according to the standard IEC 61400-12-1 [9], the calibration of a cup-anemometer should be accomplished at low turbulence conditions (axial turbulence below 2%). For the wind speeds tested [4 m/s to 16 m/s], the Reynolds number goes from 1.015 × 10^5^ to 4.063 × 10^5^. Although turbulence and density variations influence in-field measurements [3,22], since this work presents a calibration procedure, we only considered wind tunnel measures at low turbulence levels.

**Figure 4:**
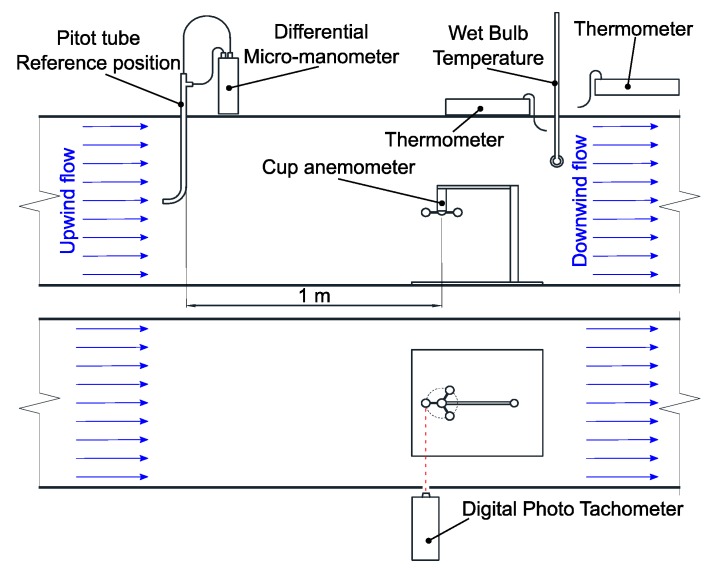
Schematic diagram of the experimental setup. Upper: front view. Lower: top view.

The standard reference device in this work is a Pitot tube: Testo stainless steel, 500 mm length, reference 06352045 and Pitot tube factor = 1. It is connected to a Testo 512 2hPa differential micro-manometer, with 0.1 Pa of resolution, a precision of ±0.01 hPa in a measuring range from 2 to 17.5 m/s, and it is capable to provide two lectures per second. The pitot tube is placed upstream at the same height as the cups, as shown in Figure 4. 

A digital Photo Tachometer model RM-1501, with a sampling frequency of 1 Hz and a precision of ±0.2 rpm, is used to obtain the anemometer rotation frequency. In order to determine the air conditions, three thermometers are used: a Tecpel 305 placed inside the tunnel to obtain the dry bulb temperature, another Tecpel 305 placed outside the tunnel to obtain the ambient temperature and finally, one alcohol thermometer placed inside the tunnel to obtain the wet bulb temperature. The setup of these elements is schematically shown in Figure 4.

The barometer placed at the roof of the Higher Polytechnic School building of the University of Jaén is used to obtain the ambient barometric pressure. These results are published on the MATRAS webpage [23], and they have been corrected taken in account the height between the building roof and the laboratory where the wind tunnel is located, according to the standard atmosphere formulation specified in ISO 2533:1975.

The testing methodology consists on placing the cup anemometer inside the wind tunnel, testing several wind speeds in a range from 4 m/s to 16 m/s and registering the cup anemometer rotation frequency together with the dynamic pressure. A steady state operating condition is set every 0.43 m/s. This sampling interval satisfies the requirements recommended by MEASNET [13] and it is usually taken by other authors in the field of anemometers calibration [24]. For each operating condition tested, the anemometer rotation frequency is registered for at least 50 s with a sampling frequency of 1 Hz (*m_f_* = 50 measurements for each operating condition). The dynamic pressure is recorded for 10 s with a sampling frequency of 0.5 Hz (*m_p_* = 20 measurements for each operating condition). The actual value of rotation frequency and dynamic pressure for a specific operating condition is taken as the mean value. A number of *n* = 29 operating conditions were tested. About half of the operating conditions were tested by increasing wind speed (15 operating conditions in odd steps) and the rest of them by decreasing the wind speed (14 operating conditions in even steps).

The wind tunnel calibration factor *k*_c_ is determined by comparing the measurements of the Pitot tube placed at the reference position and the measurements at the anemometer position (Figure 4). This procedure allows obtaining the relationship between both measurements and its uncertainty. At the anemometer position, air flow velocity is lightly higher than at the reference position.

The Pitot tube head coefficient *C_h_* is related to the alignment accuracy of the Pitot tube with the wind flow direction inside the tunnel. The ISO 3966, about measurement of fluid flow velocity using Pitot static tubes [25], suggests a *C_h_* value. This value and its corresponding uncertainty is given in Section 3. 

On the other hand, the blockage correction factor *k_f_* quantifies the influence of the anemometer shape on the mean flow field velocity *V_b_*. This correction factor is calculated through Maskell theorem [26]:

(4) $$k_{f}=\frac{V_{b}}{V}=1+\;\frac{1}{2}\;a\cdot c_{f}\cdot b,$$

where the blockage ratio *b* (anemometer front area divided by tunnel cross section) is equal to 0.025, and the product of the shape factor *a* and the force coefficient *c_f_* is *a·c_f_* = 0.5 as is recommended for unusual shapes tested within a wind tunnel [27]. The value of *k_f_* has been checked in the wind tunnel by comparing the measurements of the Pitot tube placed at the anemometer section with and without the cup anemometer inside the tunnel.

The wind dynamic pressure at the anemometer position for a *k* operating condition Δ*p_k_* is determined as the mean of *m_p_* = 20 measurements of the dynamic pressure provided by the Pitot tube Δ*p_i_* at the reference position, corrected by the factors previously discussed:

(5) $$\Delta p_{k}=\;\frac{k_{c}}{C_{h}}\cdot \;k_{f}^{2}\cdot \frac{1}{m_{p}}\cdot \overset{m_{p}}{\underset{i=1}{\sum }}\Delta p_{i}.$$

Finally, because the anemometer acquisition system obtains *m_f_* = 50 measurements for each *k* operating condition, the anemometer rotation frequency *f_k_* is determined as follows:

(6) $$f_{k}=\frac{1}{m_{f}}\;\overset{m_{f}}{\underset{i=1}{\sum }}f_{r,i}.$$

### 2.2. Quadratic Regression Uncertainty

In order to determine if the proposed calibration improves *P_w_* predictions, the uncertainty components of *P_w_* have to be determined. This section is devoted to explain how to quantify the uncertainty of the quadratic regression between air dynamic pressure and anemometer rotation frequency (Equation (7)). Authors such as Ilic et al. [28], who measure stagnation pressure inside wind tunnel, point out the high nonlinearity of the measurement process of dynamic pressure. 

The calculation of the quadratic regression uncertainty is more complex than the linear one. In fact, the “Guide to the expression of uncertainty in measurement”(GUM) [29] only provides simplified equations for the linear case. Let the following equation be the quadratic regression for a DYP-Cup anemometer, which provides the wind dynamic pressure estimation $\hat{\Delta p}$:

(7) $$\hat{\Delta p}=y_{1}+y_{2}\cdot f_{r}+y_{3}\cdot f_{r}^{2}.$$

The quadratic regression coefficients **y** = [*y*_1,_ *y*_2,_ *y*_3_] (*y*_1_ intercept: anemometer’s offset, *y*_2_ slope and *y*_3_ curvature) are determined by the least-squared method minimizing the total of the squared residuals for *n* observations. The resulted system of equations provides the regression coefficients:

(8) $$y=S^{-1}\Delta x,$$

where:

$$S=\left[\begin{array}{ccc} n & \underset{k}{\sum }f_{r,k} & \underset{k}{\sum }f_{r,k}^{2}\\ \underset{k}{\sum }f_{r,k} & \underset{k}{\sum }f_{r,k}^{2} & \underset{k}{\sum }f_{r,k}^{3}\\ \underset{k}{\sum }f_{r,k}^{2} & \underset{k}{\sum }f_{r,k}^{3} & \underset{k}{\sum }f_{r,k}^{4} \end{array}\right]\mathrm{and}x=\left[x_{1},x_{2},x_{3}\right]=\left[\underset{k}{\sum }\Delta p_{k},\underset{k}{\sum }\left(f_{r,k}\cdot \Delta p_{k}\right),\underset{k}{\sum }\left(f_{r,k}^{2}\cdot \Delta p_{k}\right)\right]$$

Applying the law of propagation of uncertainty to Equation (7), we obtain the regression combined standard uncertainty. This uncertainty is function of the **y** coefficients’ variances and covariances.

In order to obtain the aforementioned variances and covariances, we applied the law of propagation of uncertainty to Equation (8). The authors considered this approach easier to extend to complex regressions than the conventional solution used in linear regressions, as well as simpler than the method proposed by Hilbert [30], who modified the calibration equation and then applied the law of propagation of uncertainty.

According to the GUM [29], for correlated variables, the variances and covariances for the **y** coefficients can be obtained as follows:

(9) $$u\left(y_{l},y_{m}\right)=s\left(y_{l},y_{m}\right)=\overset{3}{\underset{i=1}{\sum }}\overset{3}{\underset{j=1}{\sum }}\frac{\partial y_{l}}{\partial x_{i}}\cdot \frac{\partial y_{m}}{\partial x_{j}}\cdot u\left(x_{i},x_{j}\right),$$

where *l* and *m* go from 1 to 3.

For the **x** covariances, we used the previous Equation (9); however, the variables are the dynamic pressure observations. Thus, assuming a zero covariance between different observations, the estimation of the deviation of each observation can be determined by the regression error as follows:

(10) $$u\left(x_{l},x_{m}\right)=s\left(x_{l},x_{m}\right)=\sum_{k=1}^{n}{\left({\hat{\Delta p}}_{k}-\Delta p_{k}\right)}^{2}\cdot f_{k}^{l+m-2}.$$

Finally, the application of the “law of propagation of uncertainty” to Equation (7) yields the combined standard uncertainty for the quadratic regression. Let **C** be the covariance matrix and **F** = [$\partial \hat{\Delta p}/\partial y_{1}$, $\partial \hat{\Delta p}/\partial y_{2}$, $\partial \hat{\Delta p}/\partial y_{3}$] then:

(11) $$u^{2}\left(\hat{\Delta p}\right)=F\;C\;F^{T}=u^{2}\left(y_{1}\right)+f_{r}^{2}\cdot u^{2}\left(y_{2}\right)+f_{r}^{4}\cdot u^{2}\left(y_{3}\right)+2\cdot f_{r}\cdot \left(u\left(y_{1},y_{2}\right)+f_{r}\cdot u\left(y_{1},y_{3}\right)+f_{r}^{2}\cdot u\left(y_{2},y_{3}\right)\right).$$

### 2.3. Wind Speed and Dynamic Pressure Uncertainty

According to Annex F of IEC 61400-12-1 international standard [9], in order to obtain the measurement uncertainty of a cup anemometer to measure wind speed (conventional measurement procedure), the following equations are used (“Wind speed equations”):

(12) $$V_{s}=k_{f}\cdot \sqrt{\frac{k_{c}}{C_{h}}\;}\cdot \sqrt{\frac{2\;.\;\Delta p_{Pt}}{\rho }},$$

(13) $$\hat{V}=A\cdot \;f_{r}+B,$$

where *V_s_* is the wind speed determined by the dynamic pressure Δ*p_Pt_* measured with the Pitot tube, and $\hat{V}$ is the predicted wind speed by means of a linear regression.

In the present paper, the alternative measurement procedure is based on the following equations:

(14) $$\Delta p_{s}=\;\frac{k_{c}}{C_{h}}\cdot \;k_{f}^{2}\cdot \Delta p_{Pt},$$

The Equations (7) and (14) are the “Wind dynamic pressure equations” and can be used to calibrate cup anemometers. Equation (14) is a generalization of Equation (5). Δ*p_Pt_* and Δ*p_s_* are the dynamic pressures measured with the Pitot tube at the reference position and at the anemometer position, respectively.

In order to obtain the combined uncertainty for wind speed and dynamic pressure measurements, the standard uncertainty and the sensibility coefficient for each parameter included in the calibration equations have to be computed according to Table 1.

The moist air density is obtained by means of the equation proposed in Annex F of IEC 61400-12-1 international standard [9]:

(15) $$\rho =\frac{1}{T}\left(\frac{P_{B}\cdot M_{a}}{R}-\emptyset \cdot P_{vs}\left(\frac{M_{a}}{R}-\frac{M_{v}}{R}\right)\right),$$

(16) $$P_{vs}=0.0000205\;e^{0.0631846\cdot T},$$

where $P_{B}$ is the barometric pressure (Pa), *T* is the absolute dry bulb temperature (K), *R* is the ideal gas constant (J/mol·K), *P_vs_* is the saturated vapour pressure (Pa), *M_a_* is the air molar mass, and *M_v_* is the water vapour molar mass (kg/mol). The relative humidity ∅ is determined by applying the following equation:

(17) $$\emptyset =\frac{P_{vs}\left(T_{v}\right)-A\cdot P\cdot \left(T-T_{v}\right)}{P_{vs}\left(T\right)}=\frac{P_{vs}\left(T_{v}\right)-A_{0}\cdot \left(1+0.00115\cdot (T_{v}-273.15\right))\cdot P\cdot \left(T-T_{v}\right)}{P_{vs}\left(T\right)},$$

where *T_v_* is the absolute wet bulb temperature (K), A is the so-called psychometric constant pertinent to the standard wet-bulb temperature, $A_{0}$ is the so-called psychometric constant pertinent to the standard wet-bulb temperature of 0 °C, which was experimentally adjusted by Ferrel [31]. Therefore, the moist air density can be determined via barometric pressure, dry and wet bulb temperatures, as shown in the experimental set up description. A similar procedure to that described by Picard et al. [32] was applied to the last three equations for determining the moist air density uncertainty u(ρ). The determination of the moist air dynamic viscosity is required to assess the Reynolds number in the wind tunnel. The viscosity can be calculated using the formulation recommended by Zuckerwar and Meredith [33] for air applications considering atmospheric pressure, a temperature between 10 °C and 50 °C, and a relative humidity in the range from 0.3% to 92%.

Regarding the conventional linear regression (Equation (13)), the slope *A* and offset *B* variances and covariances can be obtained via the simplified formulation proposed by the GUM [29]. The quadratic formulation presented in this work (described in Section 2.2) can be used, as well, to assess the linear regression by replacing *y_2_* by *A*, *y_1_* by *B*, and Δ*p_k_* by *V_k_,* and making *y_3_* null. In the present work, both methods, the conventional linear regression and the quadratic formulation, were applied and the results show similar values.

Finally, the squared combined uncertainty of the calibration based on “Wind speed Equations (12) and (13)” (conventional measurement procedure) was obtained taking into account the terms of the second and third columns of Table 1.

(18) $$u_{c}^{2}\left(V\right)=u^{2}\left(k_{f}\right)\cdot {\left(\frac{\partial V_{s}}{\partial k_{f}}\right)}^{2}+u^{2}\left(k_{c}\right)\cdot {\left(\frac{\partial V_{s}}{\partial k_{c}}\right)}^{2}+u^{2}\left(C_{h}\right)\cdot {\left(\frac{\partial V_{s}}{\partial C_{h}}\right)}^{2}+u^{2}\left(\rho \right)\cdot {\left(\frac{\partial V_{s}}{\partial \rho }\right)}^{2}+u^{2}\left(\Delta p_{Pt}\right)\;\cdot {\left(\frac{\partial V_{s}}{\partial \Delta p_{Pt}}\right)}^{2}+2\cdot u^{2}\left(\hat{V}\right).$$

The squared combined uncertainty of the wind dynamic pressure measurements Δ*p* based on Equations (7) and (14) (alternative measurement procedure) was obtained taking into account the terms of the second and fourth columns of Table 1.

(19) $$u_{c}^{2}\left(\Delta p\right)=u^{2}\left(k_{f}\right)\cdot {\left(\frac{\partial \Delta p_{s}}{\partial k_{f}}\right)}^{2}+u^{2}\left(k_{c}\right)\cdot {\left(\frac{\partial \Delta p_{s}}{\partial k_{c}}\right)}^{2}+u^{2}\left(C_{h}\right)\cdot {\left(\frac{\partial \Delta p_{s}}{\partial C_{h}}\right)}^{2}+u^{2}\left(\Delta p_{Pt}\right)\cdot {\left(\frac{\partial \Delta p_{s}}{\partial \Delta p_{Pt}}\right)}^{2}+2\cdot u^{2}\left(\hat{\Delta p}\right).$$

In the last two equations, the regression uncertainty applies twice; one by the calibration process and another one for in-field measurements.

### 2.4. Wind Power Density Uncertainty

The wind power density can be obtained by means of the air density and wind speed measurements (conventional method) or by means of wind dynamic pressure and wind speed measurements (alternative method, Equation (3)). Note that although both equations estimate *P_w_*, they have different uncertainties (Table 2).

The conventional procedure to estimate the wind power density with an “AS-Cup anemometer” requires to specify ρ: the air density measured by the user where the turbine is located. In the present study, a standard uncertainty of ±0.03 kg/m^3^ for the user air density is considered. The IEC 61400-12-1 international standard [9] recommends the air density normalization for power measurements, in an effort to compensate for the atmospheric variations of air density at the wind turbine location. During AEP assessment, a measurement campaign was performed, which included the measurement of air density in the range from 4 m/s to 16 m/s (at least 30 measurements must be recorded in each step of 0.5 m/s). Each air density measure is the mean value of those observations obtained in a continuous interval of 10 minutes and keeps a linear relationship with the turbine power output during that interval. The normalization determines the output power during each interval of 10 minutes considering a reference air density. The IEC standard proposes as the reference air density the mean value of all air density measures collected during the measurement campaign, or alternatively, a nominal air density pre-defined for the location.

The previous version of the IEC standard establishes that the normalization is not necessary if the actual average value of air density is within the reference density ±0.05 kg/m^3^, so that a value of ±0.03 kg/m^3^ (that we used in *P_w_* examples) means a density measure with low uncertainty, and therefore, it is a favorable situation for the conventional measurement method.

The squared combined uncertainty of the wind power density measured with an AS-Cup anemometer is:

(20) uAS2(Pw)=u2(ρ)·14V6+uc2(V)·94·ρ2·V4,

On the other hand, the squared combined uncertainty of the wind power density measured with a DYP-Cup anemometer is:

(21) $$u_{DYP}^{2}\left(P_{w}\right)=u_{c}^{2}\left(V\right)\cdot \Delta p^{2}+u_{c}^{2}\left(\Delta p\right)\cdot \;V^{2}.$$

## 3. Results and Discussion

This paper proposes a novel calibration for a cup-anemometer based on a quadratic regression. Firstly, this Section discusses how good the regression is compared with that of a conventional calibration (Section 3.1). Secondly, it shows the uncertainty for the Δ*p* and *V* estimations obtained with the proposed and the conventional calibration, respectively (Section 3.2). Finally, we demonstrate that a double calibration leads to an uncertainty reduction in *P_w_* measurements, as density does not appear in the proposed indirect measurement procedure (Section 3.3). All computations showed in this Section have been performed using Excel^®^ and the scientific computational software Mathematica^®^ in a 2 kernels Intel Core i7 2.9 GHz CPU.

The calibration process was done via 29 experimental measurements of the variables: wind dynamic pressure and cup anemometer rotation frequency. To correct the dynamic pressure lectures obtained with the Pitot tube, the following parameters were defined according the IEC 61400-12-1 international standard [9] recommendations:

$$k_{f}=1.00625\;u\left(k_{f}\right)=2.4\;10^{-4};\;k_{c}=1.004\;u(k_{c})=7\;10^{-4};\;C_{h}=0.997\;u\left(C_{h}\right)=10^{-3}$$

In order to compare the new measurement method with the conventional one, it is necessary to determine the wind speed corresponding to every wind dynamic pressure value. To reach this target, the last three parameters are used in the Equation (12), where the air density measured within the wind tunnel was obtained during the experiment from ambient temperatures measures and the ambient pressure, resulting in: $\rho =1.168\;\mathrm{kg}/m^{3}$;$\;u\left(\rho \right)=1.45\;\times \;10^{-3}\;\mathrm{kg}/m^{3}$. The air dynamic viscosity measured within the wind tunnel was obtained as well: μ =1.84 × 10^-5^ kg/m·s. It is noteworthy that the results take into account the previously defined air density value and a wind speed of *V* ∈ [4 m/s, 16 m/s], recommended in the annex F of the IEC 61400-12-1 international standard [9].

We assumed a constant air density in our proposed calibration. The reason for this assumption is that the standard about anemometers [9] only requires the density value during calibration, and we were focused on the calibration procedure.

For in-field *P_w_* measures, it is normal practice to consider a constant density value, as this simplifies the measurement procedure and the proposed approach. Because of that, we also assumed a constant density for the *P_w_* examples showed in this Section. This assumption helps to understand the advantages of the proposed double calibration; however, note that recent studies, such as the work of Ulazia et al. [34], have warned about the influence of density variations in field *P_w_* assessment. Since the proposed calibration has been tested for one density condition, further works could extend it to the case of a variable density, helping to understand its influence in field *P_w_* measurements.

### 3.1. Cup Anemometer Regression: Quadratic vs Linear

While wind speed and cup anemometer rotation frequency are linearly related, dynamic pressure and rotation frequency has a quadratic relation. An ANOVA study of the experimental data suggests that a quadratic polynomial calibration curve is the best solution to fit the wind dynamic pressure as a function of the anemometer rotation frequency. 

**Figure 5:**
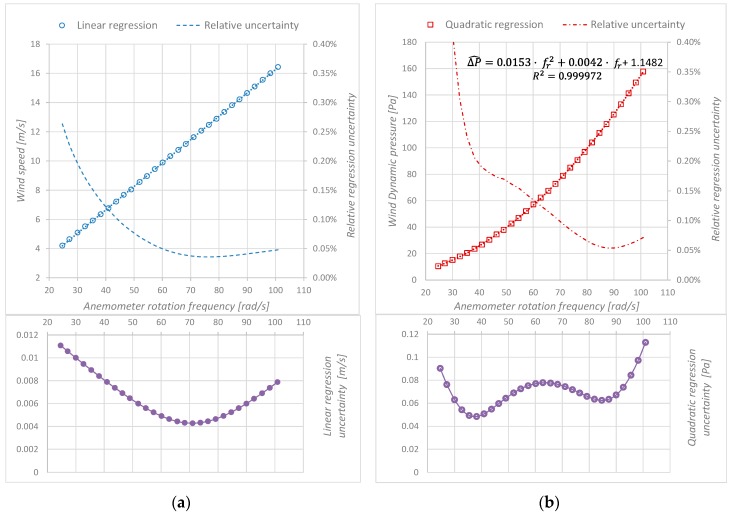
Regression uncertainty: (**a**) Linear regression between “Wind speed” and “Anemometer rotation frequency” (AS-Cup anemometer measurement method), (**b**) Quadratic regression between “Wind dynamic pressure” and “Anemometer rotation frequency” (DYP-Cup anememometer measurement method).

The idea is to compare the quadratic regression to the linear regression. To reach this target, we compute the correlation coefficients and the uncertainty of both regressions. The same 29 experimental measurements were assessed for both regressions. The correlation coefficient *r* is greater than 0.99995 for both measurement methods (Figure 5), which is the limit established by the annex F of the IEC 61400-12-1 international standard [9] for a high-quality regression.

**Figure 6:**
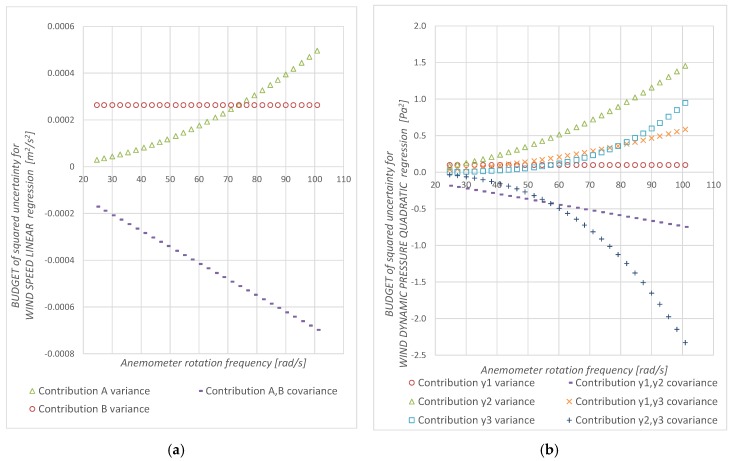
Regression uncertainty budget. (**a**) Linear regression (AS-Cup anemometer measurement method), (**b**) quadratic regression (DYP-Cup anemometer measurement method).

For both methods, the values of relative uncertainty are lower than 0.4% and decrease to 0.05% at the maximum rotational frequency tested. The DYP-cup anemometer shows higher relative uncertainty at low rotational frequency (0.4% DYP vs 0.2% AS at 30 rad/s). On the other hand, regression uncertainty curves are not monotone functions: they decrease at low *f_r_* and grow at high *f_r_*. The differences observed in the regression uncertainty shape are consequence of the variance and covariance contributions (Figure 6).

### 3.2. Wind Dynamic Pressure and Wind Speed Uncertainty

**Figure 7:**
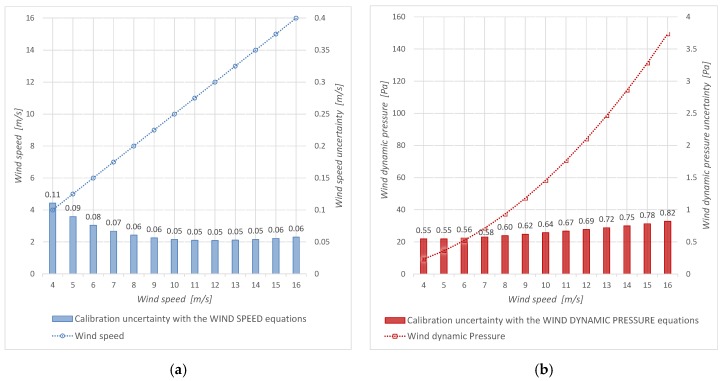
Measurement uncertainty: (**a**) “Wind speed equations” (AS-Cup anemometer measurement method), (**b**) “Wind dynamic pressure equations” (DYP-Cup anemometer measurement method).

The measurement uncertainty provided by a cup anemometer with a calibration based on the aforementioned quadratic relationship is observed in Figure 7b. The order of magnitude of the values of its relative uncertainty is similar to that obtained by means of the annex F found in the IEC 61400-12-1 international standard [9], whose graphic is shown in Figure 7a.

The calibration uncertainty for both methods (conventional and alternative) was obtained from the uncertainties combination of several parameters that appear in the applied measurement method equations. 

**Figure 8:**
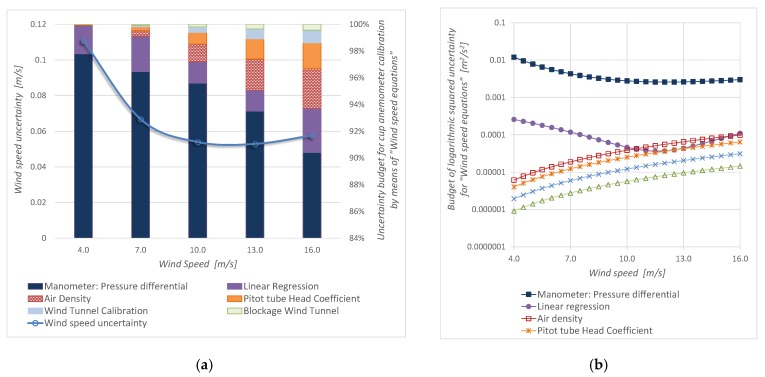
Calibration by means of “Wind speed equations” (AS-Cup anemometer measurement procedure): (**a**) wind speed uncertainty and its budget, (**b**) components of the squared uncertainty.

**Figure 9:**
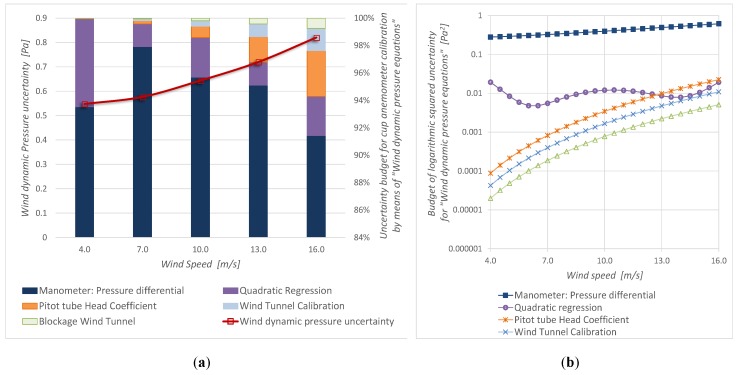
Calibration by means of “Wind dynamic pressure equations” (DYP-Cup anemometer measurement method): (**a**) wind speed uncertainty and its budget, (**b**) components of the squared uncertainty.

Figure 8 shows the parameters uncertainty for “Wind speed equations”, and Figure 9 shows the parameters uncertainty for “Wind dynamic pressure equations”. It is observed that, in both cases, the manometer uncertainty (uncertainty of the standard: *u*(Δ*p_Pt_*)) is the main contribution.

Figure 8a and Figure 9a show the calibration uncertainty budget in percentage. Figure 8b and Figure 9b portrait the specific budget in logarithmic scale and allow knowing how each uncertainty component changes with the wind velocity. 

The evolution of the relative uncertainty of each parameter that contributes to the calibrations is similar (Figure 8b and Figure 9b), except for the case of density, which only appears in the conventional measurement procedure. Density contribution is less than 3% inside the wind speed measurement interval. It must be noted that all these uncertainties are computed considering that the air density value during the user measurement process is the same as the one in the calibration process. In other words, a data normalization is required.

Inside the wind speed measurement range, it is important to note that the regression uncertainty is less than 3% for the AS-Cup anemometer measurement method and it is less than 6% for the DYP-Cup anemometer measurement method. Because the correlation coefficient is greater than 0.99995, the contribution of the regression to the calibration uncertainty is less than that of the standard device.

### 3.3. Power Density Uncertainty: Alternative and Conventional Procedures

In the present section, we are going to determine the combined uncertainty of the indirect measure of the air power density for both methods under consideration (AS-Cup anemometer and DYP-Cup anemometer). Once the calibration uncertainty is known, it is possible to compare the effect of using the DYP-Cup anemometer (Equation (3)) to determine the wind power density *P_w_*, respect to the conventional computation based on air density and wind speed (*P_w_* = 0.5·*ρ*·*V*^3^). 

**Figure 10:**
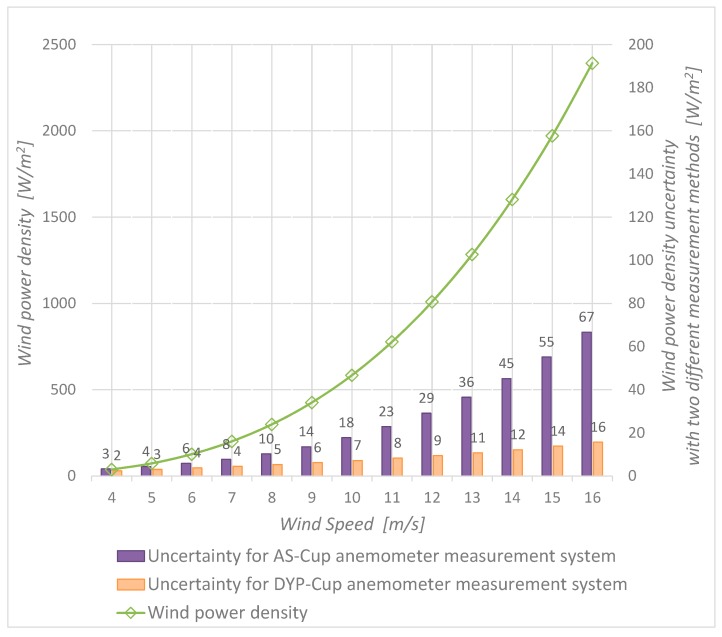
Wind power density uncertainty by means of the alternative and the conventional method.

According to Figure 10, the ratio of *P_w_* uncertainty obtained with an AS-Cup anemometer to the *P_w_* uncertainty obtained with a DYP-Cup anemometer evolves linearly with the air speed by a factor of 1/4. Let the density uncertainty be the main factor in Equation (20), and let the dynamic pressure uncertainty be the main factor in Equation (21) then, $u_{AS}^{2}≃u^{2}\left(\rho \right)\cdot \frac{1}{4}\cdot V^{6}$, $u_{DYP}^{2}≃u_{c}^{2}\left(\Delta p\right)\cdot V^{2}$, where *u*(*ρ*) = 0.03 kg/m^3^ and $u\left(\Delta p\right)=0.06\cdot V^{2}\;\;\mathrm{Pa}$. Therefore, $u_{AS}/u_{DYP}≃V/4$, which means that $u_{AS}$ is two times higher for 8 m/s, three times higher for 12 m/s and four times higher for 16 m/s than $u_{DYP}$. We conclude that, in general, $u_{AS}$ ~ $V^{3}$ and $u_{DYP}$ ~ $V^{2}$; thus, the DYP-Cup anemometer provides less uncertainty than the AS-Cup anemometer.

When wind power in a field site is determined using the conventional method, it is necessary to determine the air density at that specific location, “user air density”. In the present work, this value is considered equal to the air density during the wind tunnel tests; however, the user air density uncertainty is greater, as is explained in Section 2.4. Therefore, $\rho =1.168\;\mathrm{kg}/m^{3}$ with $u\left(\rho \right)=0.03\;\mathrm{kg}/m^{3}$.

The magnitude of the sensitivity coefficients (mainly the sensitivity coefficient of density) is the reason for the large difference, in terms of the squared uncertainty, between the *P_w_* measurement procedure based on a conventional calibration and the alternative procedure based on a double calibration. A system based on a conventional calibration requires the computation of the user air density uncertainty (Table 2 and Equation (20)), whose sensitivity coefficient grows rapidly with air speed because it is a function of wind speed raised to the third power (sixth power in a squared uncertainty budget).

**Figure 11:**
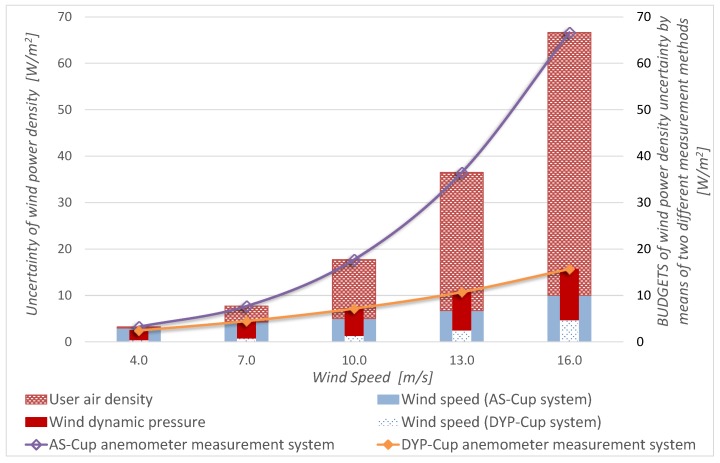
Power density uncertainty budget by means of “power density equations based on wind speed” (AS-Cup, conventional method) and “power density equations based on wind dynamic pressure” (DYP-Cup, alternative method).

Figure 11 displays *P_w_* uncertainty desegregated to evaluate the parameters contribution using the conventional and the proposed estimation procedure. It is noteworthy that density rapidly becomes the highest contribution to uncertainty with the AS-cup anemometer, while dynamic pressure contribution slowly decreases for the DYP-cup anemometer. This result points out that, even without other influence variables, density plays a key role in *P_w_* measurements and explains the uncertainty reduction with the proposed approach. Moreover, this result could support future works to focus on other phenomena that appear in field measurements, such as turbulence.

The alternative procedure provides a chance to improve energy production predictions of wind farms, which supports the recommendations of Tindal et al. [35], as they foster researchers to validate advanced measurement techniques, since they observed, for a data base of 156 wind farms, that the average actual production was 93.3 % of pre-construction projections.

## 4. Conclusions

This work shows an alternative calibration for cup-anemometers that reduces the uncertainty of the wind power density measurements. We demonstrated that a cup anemometer can measure dynamic pressure as well as wind speed via a double calibration. Therefore, unlike the conventional method, which calculates the wind power density as a function of air density and wind speed (*P_w_* = 0.5·ρ·*V*^3^), the double calibration provides the wind power density as a function of dynamic pressure and wind speed (*P_w_* = Δ*p*·*V*) obtained from the anemometer rotation frequency. The main advantage of this measurement method is that the wind power density equation does not require measuring the moist air density (considering the repeatability condition of measurement). 

The relation of dynamic pressure and rotation frequency Δ*p*(*f_r_*) follows a quadratic function: “dynamic pressure calibration”; to determine its uncertainty, it is required to assess the uncertainty of a quadratic regression. While the uncertainty computation of a linear regression, such as that used in the “wind speed calibration”, is described in the “Guide to the expression of uncertainty in measurement”, this is not the case of the quadratic one. In order to facilitate the reproducibility of the proposed method, the computation of the uncertainty for a second order polynomial has been included in detail. We have demonstrated that the relative uncertainty values of the linear and quadratic regressions are similar (Figure 5).

The uncertainty components common to the wind speed measurements based on conventional calibration and the dynamic pressure measurements based on the proposed calibration are similar. The regression uncertainties represent less than 6% of the uncertainty budget, although they depend on the quality of the regression. In this study, the correlation coefficients are greater than 0.99995. The manometer uncertainty (Pitot tube, which is the calibration standard) used to measure the difference between total and static pressure, represents more than 90% in the calibration uncertainty budget, regardless of the measurement system used for the calibration process. 

Once the double cup anemometer calibration is accomplished, the product of Δ*p* and *V* provides the wind power density *P_w_*. The new measurement procedure does not need any cup anemometer modification. Double calibration can be done in the same wind tunnel. Knowing the calibration functions for Δ*p* and *V*, in-field measurements only need the anemometer rotation frequency determination. This result can be useful to improve the energy production predictions of wind farms.

Noteworthy is the high reduction of power density uncertainty addressed by the alternative measurement method. For those applications where a cup anemometer is used to measure wind power, the proposed indirect measurement method provides less uncertainty than the conventional one. The results showed that *P_w_* uncertainty for an AS-cup anemometer is *V*/4 times the *P_w_* uncertainty obtained with a DYP-Cup anemometer, so that above 4 m/s the proposed method provides lower uncertainty than the conventional one. Note that, while the alternative measurement method does not require density uncertainty estimation, the conventional procedure does.

The results showed in Section 3 highlight that density has an important contribution in *P_w_* uncertainty. *P_w_* estimation based on the proposed double calibration could reduce this uncertainty and transfer this drawback from in-field measurements (with different density conditions than those during calibration) to the lab calibration. Density could implicitly influence the proposed calibration and, in this sense, future studies should aim to extend the proposed method to different density conditions, since the present study assumes a no-significant density variation.

Additional work could be based on the comparison of the actual power provided by a wind turbine to the estimated power provided by the new calibration procedure, as well as by the conventional calibration method. It will be interesting to take into consideration several factors that are not involved in the calibration procedure, since they can significantly influence the results, such as wind farm density and in-field turbulence.

## Figures and Tables

**Table 1:** Standard uncertainty and sensibility coefficients for each parameter included in the calibration equations.

Parameters	Standard Uncertainty	Sensibility Coefficients: Equation (12)	Sensibility Coefficients: Equation (14)
Blockage correction factor (*k_f_*)	$u\left(k_{f}\right)$	$\partial V_{s}/\partial k_{f}$	$\partial \Delta p_{s}/\partial k_{f}$
Wind tunnel calibration factor (*k_c_*)	$u\left(k_{c}\right)$	$\partial V_{s}/\partial k_{c}$	$\partial \Delta p_{s}/\partial k_{c}$
Pitot tube head coefficient (*C_h_*)	$u\left(C_{h}\right)$	$\partial V_{s}/\partial C_{h}$	$\partial \Delta p_{s}/\partial C_{h}$
Air density (*ρ*)	*u*(*ρ*)	$\partial V_{s}/\partial \rho$	
Wind Dynamic pressure (Pitot tube) ($\Delta p_{Pt}$)	$u\left(\Delta p_{Pt}\right)$	$\partial V_{s}/\partial \Delta p_{Pt}$	$\partial \Delta p_{s}/\partial \Delta p_{Pt}$
Linear regression (*A, B*)	$u_{lr}\left(V\right)=$ $\sqrt{u^{2}\left(B\right)+f_{r}{}^{2}\cdot u^{2}\left(A\right)+2f_{r}\cdot \;u\left(A,B\right)}\;$	-	-
Quadratic regression (*y_1_, y_2_, y_3_)*	$u\left(\hat{\Delta p}\right)\;\;$ Equation (11)	-	-

Note: *k_f_*, *k_c_*, *C_h_* values and their uncertainties are provided in Section 3.

**Table 2:** Standard uncertainty and sensibility coefficient of the wind power density equations.

Parameters		Standard Uncertainty	Sensibility Coefficients: Equation (20)	Sensibility Coefficients: Equation (3)
User air density	ρ	u(ρ)	$\partial P_{w}/\partial \rho =1/2\;\;V^{3}$	
Wind speed	*V*	$u_{c}\left(V\right)$	$\partial P_{w}/\partial V=3/2\;\rho \;V^{2}$	$\partial P_{w}/\partial V$= Δp
Anemometer dynamic pressure	Δp	$u_{c}\;\left(\Delta p\right)$		$\partial P_{w}/\partial \Delta p=\;V$

## Nomenclature

A	Slope for linear regression [m]
a	Factor depending on the shape of the structure within the wind tunnel
B	Intercept or offset for linear regression [m/s]
b	Blockage ratio
$c_{f}$	Force coefficient of the structure within the wind tunnel
$C_{h}$	Pitot tube head coefficient
$C_{P}$	Power coefficient
$f_{r}$	Anemometer rotation frequency [rad/s]
$k_{c}$	Wind tunnel calibration factor
$k_{f}$	Blockage correction factor
$m_{f}$	Number of measurements of $f_{ri}$ for each operating condition
$m_{p}$	Number of measurements of $\Delta p_{i}$ for each operating condition
$M_{a}$	Air molar mass [kg/mol]
$M_{v}$	Water vapour molar mass [kg/mol]
n	Total number of operating conditions
P	Electrical power [W]
$P_{B}$	Barometric pressure [Pa]
$p_{static}$	Static pressure fluid flow [Pa]
$p_{total}$	Total pressure fluid flow [Pa]
$P_{vs}$	Saturated vapour pressure [Pa]
$P_{w}$	Wind power density [W/m^2^]
R	Ideal gas constant [J/(mol·K)]
r	Correlation coefficient
$R^{2}$	Coefficient of determination
S	Blade swept area [m^2^]
T	Absolute dry bulb temperature [K]
*T_v_*	Absolute wet bulb temperature [K]
u	Standard uncertainty
$u_{c}$	Combined standard uncertainty
V	Wind speed [m/s]
$\hat{V}$	Wind speed predicted via linear regression [m/s]
$V_{b}$	Mean flow field velocity inside wind tunnel, with blockage effect [m/s]
$V_{s}$	Wind speed determined by the Pitot tube [m/s]
$x_{1}$	$\underset{k}{\sum }\Delta p_{k}$ the first variable change to assess the quadratic regression uncertainty [Pa]
$x_{2}$	$\underset{k}{\sum }(f_{k}\cdot \Delta p_{k})$ the second variable change to assess the quadratic regression uncertainty [Pa/s]
$x_{3}$	$\underset{k}{\sum }\left(f_{k}^{2}.\Delta p_{k}\right)$ the third variable change to assess the quadratic regression uncertainty [Pa/s^2^]
$y_{1}$	Intercept or offset for quadratic regression [Pa]
$y_{2}$	Slope for quadratic regression [Pa·s]
$y_{3}$	Curvature for quadratic regression [Pa·s^2^]
Δp	Wind dynamic pressure. Fluid kinetic energy per volume unit [Pa]
$\hat{\Delta p}$	Wind dynamic pressure predicted via quadratic regression [Pa]
$\Delta p_{Pt}$	Wind dynamic pressure function measured with the Pitot tube at the reference position [Pa]
$\Delta p_{s}$	Wind dynamic pressure determined by the Pitot tube [Pa]
ρ	Moist air density [kg/m^3^]
∅	Relative humidity
ψ and ϑ	Ferrel coefficients
